# Buffalo Milk: Alternative Use for Soap Preparation Enriched with Vegetables

**DOI:** 10.3390/molecules31040734

**Published:** 2026-02-20

**Authors:** Barbara la Gatta, Flavia Dilucia, Maria Teresa Liberatore, Mariacinzia Rutigliano, Aldo Di Luccia, Marzia Albenzio, Mariangela Caroprese

**Affiliations:** Department of Sciences of Agriculture, Food, Natural Resources and Engineering (DAFNE), University of Foggia, Via Napoli, 25, 71122 Foggia, Italy; flavia.dilucia@unifg.it (F.D.); mariateresa.liberatore@unifg.it (M.T.L.); mariacinzia.rutigliano@unifg.it (M.R.); adiluccia@gmail.com (A.D.L.); marzia.albenzio@unifg.it (M.A.); mariangela.caroprese@unifg.it (M.C.)

**Keywords:** buffalo milk, artisanal soaps, natural product, bioactive compounds, polyphenols

## Abstract

The surplus in the production of buffalo milk determines the possibility of finding alternative solutions for its use. Indeed, the utilization of milk in cosmetic formulations has been met with great approval by consumers, primarily due to its highly appreciated emollient characteristics. The aim of this research was to test an alternative use of buffalo milk in the production of artisanal solid soaps, using buffalo milk as raw material and Lavender, Thyme, and Grape pomace as sources of natural bioactive compounds. The analytical approach was focused on using vegetable materials in three forms: fresh, dried, and freeze-dried. For this purpose, the chemical features of both raw materials and artisanal soaps were determined in order to understand the feasibility of these productions. All formulated artisanal soaps revealed good chemical characteristics, such as a low moisture content, and got high scores in the sensory evaluation, with those with Lavender and Grape pomace being the most appreciated formulations. Furthermore, adding vegetable materials increased the bioactive molecules content, as demonstrated by the data obtained from total polyphenol content and antioxidant activity. Therefore, the addition of plants and vegetables to the formulation could represent an innovative production of natural soaps and be a further element for the market trends.

## 1. Introduction

Buffalo milk is commonly used for the preparation of different cheeses, but it is recognized worldwide for the production of PDO Buffalo mozzarella cheese. The seasonal mismatch between the delivery of Italian Mediterranean buffalo and the dairy market demand for mozzarella cheese led producers to freeze the milk in winter to be used in summer, causing a milk surplus [1]. The utilization of frozen buffalo milk in food preparations is frequently the subject of contention regarding the fresh and chemical qualities of the products. Consequently, the potential for employing it in cosmetic preparations has been assessed as a valid alternative. Therefore, a possible solution for the use of the remaining milk could be its utilization as a natural ingredient in other productions. Milk is one of the ingredients used in the cosmetic world, thanks to its properties that have a positive effect on the skin [2]. Milk contains proteins and fats, whose functional properties make milk soap effective in moisturizing and nourishing the skin [3,4,5]. Moreover, it is perceived as a natural, skin-healthy product by the consumers. Susanti et al. [6] and Murti et al. [7], to resolve the short storability and easy perishability of milk, proposed an alternative product, such as milk soap bar, studying an appropriate formulation, based on vegetable oils and fresh cow and goat milk. They paid particular attention to the physico-chemical characteristics and acceptability of milk soap at different storage times to obtain the best features, as well as its safety for human skin. However, buffalo milk can also have a nourishing effect on the skin due to its composition. In fact, buffalo milk has higher levels of fat, protein, vitamins A and C, along with the presence of the blue-green pigment (biliverdin), as well as a bioactive pentasaccharide and gangliosides, which are not present in cow milk [8]. The decision to utilize such a noble product in the production of soaps stems from the significance of this cosmetic product for human health. From ancient civilizations to the present day, the use of soap has contributed to improving human health through personal hygiene care and preventing diseases. The basics of soap making have not fundamentally changed over the years, with the process traditionally consisting of animal fats’ saponification reaction and alkali hydrolysis, resulting in a blend of sodium or potassium salts of the long-chain fatty acids. Potassium-based soap creates a more water-soluble product than sodium-based soap and, therefore, it is called “soft soap”. The modern soap preparation requires the addition of perfumes for scenting, dyes for coloration, and air to be blown to make the soap float [9,10]. In recent years, considering the negative effects of synthetic surfactants on the skin lipid layer, the need to produce a more respectful and sustainable product for the skin and environment arose, since consumers started to pay attention to the use of synthetic oils in the production of cosmetics. Actually, consumers started to look after their skin health, mainly for hydration, preferring the use of essential oils or plant extracts in order to preserve the lipid layer of the skin. Therefore, contemporary soaps are produced with particular attention to the use of natural ingredients [11,12]. Moreover, natural ingredients that are rich in bioactive compounds can be recovered from vegetable by-products, as well as from plants and fruits [13]. In this context, for example, these authors presented a green perspective, based on the reuse of waste materials such as almond shells and orange peel, and by using cooking oil to manufacture soap, to contribute to the development of useful waste management with an ecological approach.

In this study, buffalo milk samples were mixed with Lavender flowers (*Lavandula angustifolia*), Thyme plant (*Thymus citrodorius*), and Grape pomace, with recognized pharmacological properties, in order to determine the appropriate milk saponification reaction and the best formulation through the mixing with vegetable oils. Finally, chemical parameters, polyphenols, antioxidant activity, and sensorial attributes of the prepared soaps were evaluated.

## 2. Results and Discussion

### 2.1. Chemical Characteristic of Raw Materials

The chemical characteristics of the raw material were evaluated with the objective of ascertaining its initial characteristics and enhancing the comprehension of the characteristics of the soap obtained.

#### 2.1.1. Quality of Milk and Extra Virgin Olive Oil

The quality of extra virgin olive oil is a critical factor in the production of solid soaps, as it determines their capacity to hydrate, nourish, and soothe the skin. Low acid value in oil indicates that the oil will be stable over a long period of time and protect against rancidity and its suitability in soap making [14]. As presented in Table 1, the principal chemical characteristics must be determined in order to ascertain the commercial quality of extra virgin olive oil used in this research. As expected, all the values confirmed are within the legal limits, and the oil used is considered an extra virgin olive oil. Moreover, Table 2 shows the composition of cow and buffalo milk used.

**Figure 1:**
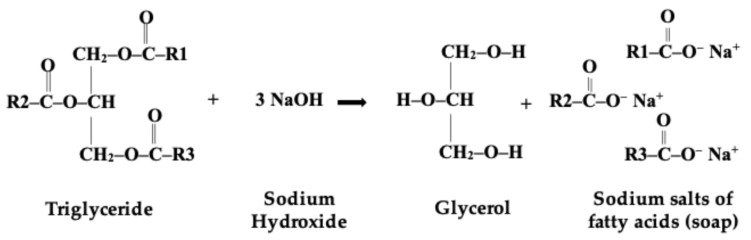
Hydrolysis of triacylglycerols and saponification reaction.

As reported in the literature [15], buffalo milk is richer in fat and proteins, caseins included, than cow milk, 7.96 vs. 4.88 and 4.62 vs. 3.77, respectively. Some milk components have cosmetic properties. Cotte [16] listed milk proteins, lactoferrin, phospholipids, and ceramides, among other important milk components. In particular, milk proteins, due to their amphipathic nature, could work as active substances for skin hydration and could also be used as natural surfactants to produce cosmetic commodities that are less aggressive, while those with a glycosylated part could be largely used in products against skin aging. Lactoferrin, which has a known high iron chelating property, could prevent bacterial proliferation and free radicals’ production after skin stress; phospholipids or ceramides could be widely used in cosmetology, either as active drugs or for obtaining systems such as liposomes, if separated and purified from the fat globule membrane. Ribeiro and Ribeiro [17] referred to the beauty characteristic of milk goat as a special product to be used for hair, skin care, and cosmetic products, while Wu et al. [5] uncovered the benefits of milk exosomes, which can be isolated on a large scale at a low cost, for skin protection, enhancing the barrier role. Finally, milk fat plays a key role in the saponification process (Figure 1), and milk fat contains triglycerides, as major components, diglycerides, mono glycerides, and fatty acids, in a similar way as extra virgin olive oil, that is rich in unsaturated fatty acids, especially oleic acid and coconut oil, and rich in saturated fatty acids, particularly lauric and myristic acids [6,18]. Therefore, according to the features of the tested raw materials (i.e., cow and buffalo milk) and based on the results obtained by the first trials of production, the enriched formulations were produced by using buffalo milk as the starting raw material.

#### 2.1.2. Chemical Characteristic of Vegetable Materials

The chemical characterization of Lavender, Thyme, and Grape pomace, differently processed, is shown in Table 3. The difference in pH value for each raw material was significant (*p* < 0.05), considering the three types of raw materials tested. Grape pomaces showed the lowest pH, ranging between 3.40 and 3.54, with respect to the plants that revealed a higher pH, ranging between 5.47 (fresh Lavender) and 6.41 (patented Thyme).

As expected, the moisture percentage was higher for fresh Grape pomace, with 82.5%, followed by fresh Lavender (63.5%) and fresh Thyme (51.7%), and accordingly, the moisture content after the application of the patented technology also followed the same order (Grape pomace > Lavender > Thyme), but reaching much lower values. The total polyphenolic content (TPC) showed low values for fresh products, while the highest values were obtained from patented products, with significant differences (*p* < 0.05). The highest values for TPC were found for patented Grape pomace, which also showed the highest antioxidant activity. It was interesting to note that, although fresh Lavender had the lowest TPC (394 mg of Gallic Acid/100 g), it exhibited a high antioxidant activity (1265 µmol TEAC/100 g), which was not significantly different from that of patented Lavender (1282 µmol TEAC/100 g). It must be considered that, studying the dried and patented products, the same trend for TPC was observed, with patented products > dried products (*p* < 0.05). On the other hand, the antioxidant activity trends were different: in the case of Grape pomace, the values of the antioxidant activities followed the same order as the TPC, whereas in the case of Thyme and Lavender, the dried products registered higher values than the patented products, which in turn, had the highest TPC values. This phenomenon was already observed by Kähkönen et al. [19] during a screening of the total phenolic content and antioxidant activity of a large number of plant material extracts, and by la Gatta et al. [20] in a study on the omelet enriched with freeze-dried vegetables. These authors highlighted that the antioxidant activity was not a direct result of the content of phenolic compounds and suggested that the phytochemical activities could be correlated to the nature of phenols contained in the extracts and to their possible interactions, which can amplify or attenuate the antioxidant potential. In addition, the antioxidant activity can be modified by the transformation processes, in accordance with the results of Nicoli et al. [21], who showed that the concentration of natural antioxidants, in the case of thermal treatments, could be affected, though the overall antioxidant properties of food products have been maintained or even improved.

**Figure 2:**
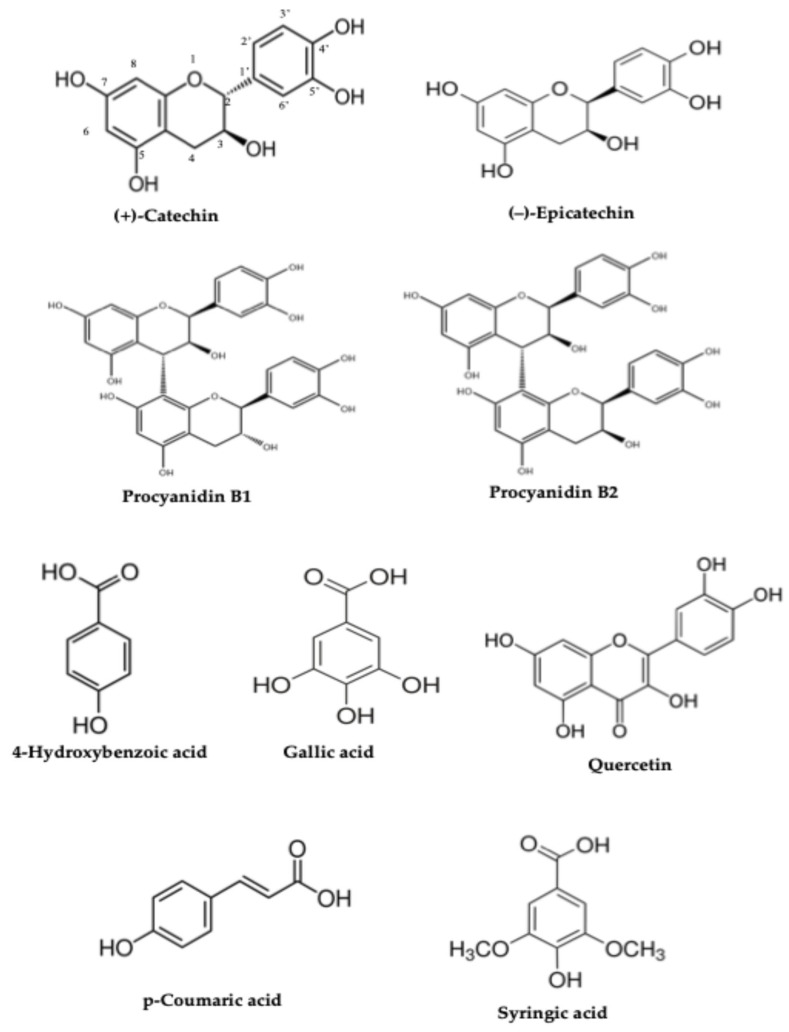
Flavonoid and phenol structures detected by reversed-phase liquid chromatography.

**Figure 3:**
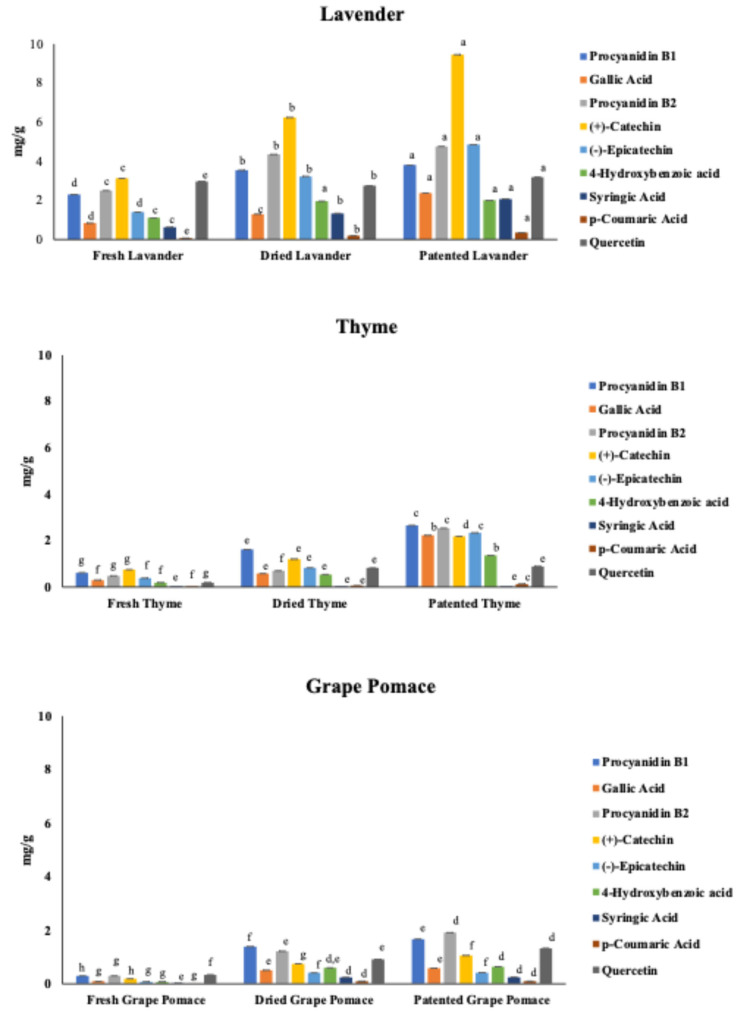
Polyphenol content in Lavender, Thyme, and Grape pomace raw materials. ^a, b, c……..h^ (*p* < 0.05). Significant differences among the detected polyphenols in the raw materials’ samples are expressed with different superscript letters.

Polyphenolic compounds detected by reversed-phase liquid chromatography (RP-HPLC) are depicted in Figure 2, and their different contributions according to the plant/vegetable, fresh, dried, or patented use are illustrated in Figure 3. The highest content of polyphenols was observed for Lavender samples, followed by Thyme and Grape pomace samples. Generally, a linear increase was observed passing from fresh to patented products, as inferred in Figure 3.

The increasing content of polyphenols passing from fresh to dried products could be explained by the moisture reduction, while the trends related to the patented products were probably due to reactions of the phenols during dehydration processes, which revealed a slower increase that was not only ascribable to the water loss. Mostly, Lavender showed an increase for each polyphenolic molecule passing from fresh to patented samples, with (+)-catechin being the most represented (Figure 3). The differences were significant for all the components (*p* < 0.05), except for 4-hydroxybenzoic acid, whose values were not significantly different for the dried and patented samples in the case of Lavender and Grape pomace. Polyphenols shown by the Thyme samples revealed an increasing trend already from fresh to dried samples, with significant differences among components (*p* < 0.05), except for syringic acid. Polyphenols of Grape pomace showed an increasing trend from fresh to dried samples, while gallic acid, (–)-epicatechin, 4-hydroxybenzoic acid, syringic acid, and p-coumaric acid did not show significant differences passing from dried to patented samples (Figure 3).

Fernández-Pachón et al. [22] studied the relationship between antioxidant activity and polyphenolic composition in red wines through solid-phase extraction. They separated three main fractions (phenolic acids, flavan-3-ol and anthocyanins, and flavonols), finding that the fraction containing flavanols and anthocyanins showed the highest antioxidant activity in every analyzed case. Gregoris & Stevanato [23], investigating the correlations between the polyphenolic composition and antioxidant activity of Venetian propolis, observed that the presence of a double bond C2—C3 conjugated with the aromatic ring could lead the hydroxyl group, linked to the C3, to undergo monoelectronic oxidation, causing the formation of a hydroxyl radical, whose unpaired electron can be delocalized in position 2 and in the ring. Indeed, they observed that a hydroxyl group at C3 increased the antioxidant activity, as in the case of Galangin and Kaempferol. The polyphenols found in the soaps were characterized by a hydroxyl group linked to the C3, and, in addition, Quercetin has both a double bond C2—C3 and a hydroxyl group linked to the C3. Considering the proportions of the polyphenols in the plants/vegetables, we can observe that Lavender had the highest content of all the quantified polyphenols, but it has been shown already that Grape pomace is characterized by the presence of resveratrol [24], a powerful antioxidant molecule, which can justify the highest antioxidant activity found in the patented sample (Table 3). Therefore, the nature of polyphenols, their interactions, and enzymatic and non-enzymatic reactions during the processing and/or storage can affect the resultant antioxidant activity.

### 2.2. Chemical Characteristics of Soaps

The chemical characterization of the different formulations of milk soaps prepared with the conventional methodology of saponification is shown in Table 4.

During daily skin cleansing, the soap’s pH can modify the normal skin pH (<5) [25,26] and the pH of the stratum corneum that ranges from values below 5 in the outer layers to approximately 7 at the interface of the viable epidermis [27]. More recently, Fukuda et al. [28] found that the stratum corneum has three distinct stepwise pH zones: middle acidic (pH 5.4), lower-moderately acidic (pH 6.0), and upper-nearly neutral (pH 6.7). They also found that several key enzymes involved in the synthesis and maintenance of a competent skin barrier are largely impacted by pH. Therefore, the pH of a soap should not be different from the pH of the skin, which ranges between 5 and 7, as suggested by Widyasanti et al. [29]. The pH values of the solid soaps produced in our experimentation (Table 4) ranged between a minimum of 10.12 (**F1**, cow milk soap) and a maximum of 10.84 (**F2**, buffalo milk soap) with significant differences (*p* < 0.05). These findings were in accordance with the literature, where the pH value of solid bar soap can vary between 8 and 10 [30,31], justifying that the production of the new formulated artisanal soap bars could be improved. It was noteworthy that the soaps manufactured with extra virgin olive oil (**F3**, 10.44 UpH), without milk and vegetables or plants, showed significant differences (*p* < 0.05) compared to **F1** and **F2** milk soaps. Considering that soap formulations from **F4** to **F12** were manufactured by using buffalo milk (i.e., based on the **F2** formulation), the presence of plants/vegetables probably mitigated the high pH value of **F2**. Although all the soap samples showed a pH value above the natural range (6.5–7.5), and after a single wash with a bar soap, the pH was higher than the normal range of 5–5.5 to 7.5, within several hours after washing, due to the buffering capacity of the skin, the pH of the skin surface gradually decreased into the normal range [32,33,34]. Furthermore, it has to be considered that our production involved only natural products and no topical irritants or additives; therefore, the tested bar soaps could mitigate the negative effect of the medium alkaline pH.

Moisture content is a parameter that can be used to assess the product’s shelf-life. From the values obtained by the analysis, the moisture varied from a minimum 3.76% of the soap containing fresh Thyme (**F7**) to a maximum 7.28% of the soap containing fresh Grape pomace (**F10**) (Table 5). Since the production procedure was the same, the significant difference (*p* < 0.05) in the moisture percentage observed between the cow milk soap (**F1**, 4.20%) and buffalo milk soap (**F2**, 6.43%) could probably be attributed to the different compositions of the raw materials (i.e., cow and buffalo milk). The Lavender and Grape pomace formulations (**F3**, **F4**, and **F5** and **F10**, **F11**, and **F12**, respectively) did not show significant differences between the formulations with the addition of fresh and dried samples (**F3** Vs **F4** and **F10** Vs **F11**, respectively), whereas the formulations with the addition of patented samples (**F5** and **F12**, respectively) showed a significant difference (*p* < 0.05). The differences in the moisture percentage for the formulations containing Thyme (i.e., **F6** (7.05%), **F7** (3.76%), and **F8** (4.75%)) appeared to be significant (*p* < 0.05) for all the considered samples. The moisture content in the milk soaps was much lower than the recommended percentage (10–15%) by the Encyclopedia of Industrial Chemical analysis [35]. The implications derived from the low moisture content in the experimental soaps would lead to a slowdown of the hydrolysis reaction and, therefore, to a likely prolonged shelf-life of the product on storage [36,37].

Insoluble fatty matter (%MIA) is a parameter used to determine the purity of soap [38]. It is the measure of non-soap ingredients, known as builders or fillers, such as sodium silicate, sodium phosphate, sodium carbonate, and minor constituents, such as bleachers, whitening agents, and fluorescing agents, in the finished product. A soap with a high MIA value suggests that it contains a high level of impurities, which may be attributed to the level of impurity of the alkali used for the soap manufacturing [38]. The values of MIA of the soaps ranged between 51.39% (**F9**) and 61.15% (**F2**), as shown in Table 4. The differences among MIA values were not significant, indicating that the observed variations were due to impurities of the similar components used in the soap manufacturing.

Totally fatty matter (%TFM) is the parameter used to describe the quality of a soap [39]. It is defined as the total amount of fatty matter, mostly fatty acids, that can be separated from a sample after splitting with a mineral acid, usually hydrochloric acid. TFM values of the formulated milk soaps ranged from 40.65% (**F7**) to 50.80% (**F10**) (Table 4). Most of the formulations did not show significant differences, whereas the differences were significant (*p* < 0.05) between **F7** (40.65%) and **F9** (50.50%) (Thyme samples). According to the International Standards (ISO 685) [40], good-quality soaps show a percentage of TFM above 76%, and they are of grade I. A percentage of TFM above 60% belongs to grade II, while a percentage of TFM above 50% belongs to grade III (the worst one) [41]. The detection of values lower than those declared by the ISO 685 could be the result of the addition to the formulation of too many additives, like fillers and preservatives, to confer special properties. However, a low TFM value could also be due to the presence of unreacted NaOH in the mixture [42]. In our case, the addition of plants and vegetables could have affected the soap quality, considering them as additives. Moreover, the %MIA values are different from those of conventional solid soaps because the use of a complex matrix like buffalo milk introduces significant quantities of non-fatty solids—specifically caseins, whey proteins, and minerals—which remain trapped within the soap structure and are recovered as insoluble matter during analysis. As reported in the literature, this parameter often serves as an indicator of non-soap constituents or additives. Similarly, the lower %TFM values are a direct consequence of the “dilution” of the fatty matter by the high concentration of milk solids and vegetable additives [42,43]. Moreover, we must also consider that soap without the addition of plants and vegetables (**F1**, **F2**, and **F3**) showed low percentage values of TFM of 45,19%, 41,73%, and 44.43%, respectively. Therefore, we could conclude that the TFM values were essentially due to unreacted NaOH or/and to a low concentration of fatty acids that lowered the milk soap quality (less than grade III).

Total alkali analysis is often used as a quality parameter because it ensures the safety and efficacy of the product by measuring the quantity of basic agents present. In general, a lower value indicates a better soap quality because an excess of alkali can cause the fats and oils naturally present on the skin to saponify, resulting in the formation of a protective coating. The analysis of the artisanal soaps produced yielded a low value. However, this value improved with the incorporation of vegetables into the formulation, particularly with the freeze-dried ones. The results shown in Table 4 agreed with the consideration of Zayed et al., 2024 [44], who demonstrated that an artisanal solid soap could be characterized by a higher value of pH and a lower value of total alkali.

The results of total polyphenolic content (TPC) and antioxidant activity assays, considering the extractions with methanol and water, are also reported in Table 4. The extraction with water was carried out in order to assess the performance and features of the artisanal soaps by testing the conditions of the normal use of this type of product. From the outcomes of the two extractions, it was observed that TPC values were greater for water extraction than for methanol, unlike the antioxidant activity values, which were consistently higher for the methanol extracts than for the water extracts. Indeed, by evaluating the range of values obtained considering a minimum and a maximum quantified, TPC values ranged from 187 to 383 (mg Gallic Acid /100 g) for methanol extracts, whereas they ranged from 383 to 765 (mg Gallic Acid /100 g) for water extracts, with significant differences among the samples (*p* < 0.05). Concerning the antioxidant activity values, they ranged from 1114 to 1573 (µmol TEAC/100 g) for methanol extracts and from 214 to 958 (µmol TEAC/100 g) for water extracts. Regarding the formulations **F1**, **F2**, and **F3**, despite the absence of plants/vegetables in the formulations, the detection of polyphenols and antioxidant activity was registered, considering that it is also well documented in milk, coconut, and olive oil by Sik et al. [45], Marina et al. [46], and Servili et al. [47]. As a matter of fact, it was found that the formulation containing olive oil showed the second-highest value related to the antioxidant activity for both methanol and water extracts, although it was registered as an intermediate value for the TPC. The highest value of antioxidant activity was shown by the formulation **F7** (fresh Thyme) with the methanol extraction, corresponding to 285 mg Gallic Acid /100 g for the TPC, whereas the highest value of antioxidant activity for the water extraction was shown by the formulation **F11** (dried Grape pomace), corresponding to 351 mg Gallic Acid /100 g for the TPC (Table 4). The soap formulations **F8**, **F9**, and **F11** did not show significant differences for the methanolic extracts related to the antioxidant activity, while **F8** and **F10** did not show any difference for the water extracts. In the formulation **F9**, no antioxidant activity was detected in the water extract, as well as in **F6** (Table 4). It was noteworthy that the TPC value for this latter formulation was quite high for both methanol and water extracts (298 and 666 mg Gallic Acid/100 g), while **F9** showed lower values (275 and 566 mg Gallic Acid /100 g). All these results highlighted a non-direct relation between the TPC values and the antioxidant activity. Actually, the **F12** formulation was an example that showed an equal value for TPC for both methanol and water extracts, although in the first case (methanol extract), the TPC value was the highest, while in the other (water extract), the registered value was very low, with remarkable differences for the related antioxidant activity.

Zain and Omar [48], comparing the antioxidant activity, total phenolic content, and total flavonoid content of water and methanol extracts of Phyllanthus species, found that the chemical contents and antioxidant activity of the methanol extract were consistently higher than those of the water extract. However, our results showed an opposite trend in the two extracts; most likely, this disagreement was due to a different dissolution of the soaps in methanol and in water, because of their different polarity, which was reflected by the phytochemical solubility. Actually, methanol is more efficient than water for the extraction of semi-polar compounds, such as flavonoid structures [49,50], and shows higher values for TPC and DPPH activity when compared to water extracts [51]. However, it must be considered that the antioxidant activity also depends on other non-polyphenolic compounds, such as vitamins, minerals, and terpenes, which, moreover, could exert a synergistic effect with TPC and total flavonoid content, enhancing the antioxidant properties [52]. All these considerations led us to assume that the nature of phytochemicals contained in the plants or vegetables, their interactions, and enzymatic and non-enzymatic reactions during the processing and/or storage can determine the resultant antioxidant activity [53,54].

**Figure 4:**
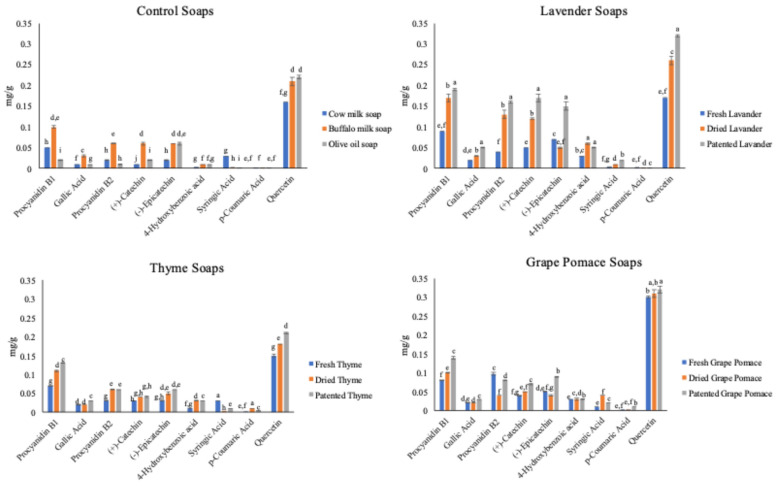
Polyphenol content in Lavender, Thyme, and Grape pomace soaps samples. ^a, b, c……..i^ (*p* < 0.05). Significant differences among the detected polyphenols in the soap samples are expressed with different superscript letters.

The results of the soap polyphenols chromatographic separation are depicted in Figure 4. Generally, the content of detected polyphenols decreased in every enriched-soap sample by about 90% with respect to the raw materials, varying in a range from a few micrograms to 350 μg. This was an expected value given the percentage of vegetable parts used in the soap’s formulation. The most represented polyphenols were procyanidin B1, procyanidin B2, (+)-catechin, and Quercetin, while those with the lowest values were syringic acid and *p*-coumaric acid (Figure 4). Considering that the coconut oil was always used in all the formulations, the variation in Quercetin content was due to the presence of cow or buffalo milk and olive oil. In fact, this polyphenol is known to be present in milk and oil, as already assessed by Gbylik-Sikorska et al. [55] and Cairone et al. [56].

The highest values in the different soap formulations were revealed in the Lavender and Grape pomace soaps, especially in the formulations containing the dried and patented ingredients, which preserved the highest percentage of polyphenols when compared with the raw materials. The variations observed for the polyphenols can be explained by both the low percentage used in the formulation and the conditions of the soap production process. In particular, the pH variations and moisture content can lead to chemical reactions and/or structural re-arrangements that can change their nature or availability, making the polyphenols no longer detectable.

### 2.3. Sensory Soaps Evaluation

**Figure 5:**
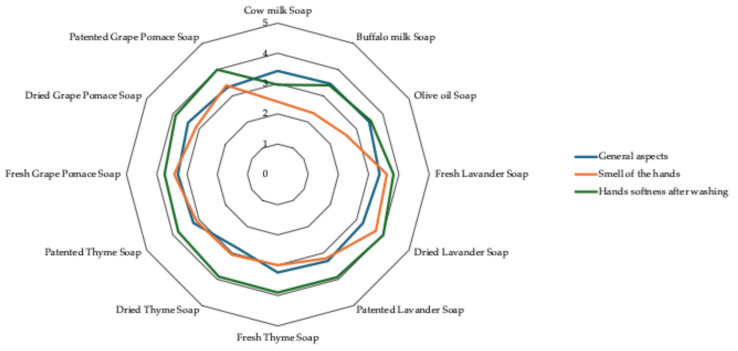
Sensory evaluation of the soaps.

A descriptive analysis of the soap formulations was carried out to test the perception-based sensory attributes: general aspect, smell of the hands, and softness after washing. The sensory evaluations of 50 people (men and women, 20–55 years old) are graphically represented in Figure 5. Concerning the general aspect, the best values were assigned to cow milk soap, buffalo milk soap, Olive oil soap, and dried Grape pomace soap, while the lowest was assigned to dried Thyme soap, although all the differences among formulations were not significant. Buffalo milk Soap had the lowest value for the smell of the hands, whereas the best values were ascribed to Lavender formulations (as fresh and dried soaps). Concerning this attribute, the soap formulations **F4**, **F5**, **F6**, **F8**, **F9**, **F10**, **F11**, and **F12** did not show significant differences among one another and got good scores. Soap formulations **F1**, **F2**, and **F3** showed significant differences (*p* < 0.05) when compared to the above-mentioned formulations, with **F2** showing the lowest score (2.74). The scores of hands softness after washing showed significant differences (*p* < 0.05) among buffalo milk and olive oil soap (**F2**, **F3**) and cow milk soap (**F1**), whereas all the formulations based on buffalo milk did not show significant differences between them and had a major score compared to the base soaps. The worst judgment was assigned to cow milk soap, whereas the soaps based on buffalo milk with plants/vegetable ingredients showed good scores, in particular dried Lavender soap and patented Grape pomace soap. Generally, the highest scores were obtained by the soap formulations **F5** (dried Lavender soap) and **F12** (patented Grape pomace soap).

## 3. Materials and Methods

### 3.1. Chemicals and Reagents

Methanol, ultra-pure water, and acetonitrile were of analytical grade and purchased from Sigma-Aldrich/Merch KGaA, (Darmstadt, Germany); procyanidin B1, gallic acid, procyanidin B2, catechin, epicatechin, 4-hydroxybenzoic acid, syringic acid, p-coumaric acid, and quercetin were of HPLC grade and purchased from Sigma-Aldrich/Merch KGaA (Darmstadt, Germany); Folin–Ciocalteu reagent, sodium carbonate (Na_2_CO_3_), 2,2-diphenyl-1-picryl-hydrazyl (DPPH), and Trolox were purchased from Sigma-Aldrich/Merck KGaA (Darmstadt, Germany); acetic acid and sodium hydroxide (NaOH) were purchased from Honeywell (Charlotte, NC, USA); and hydrochloric acid, ethanol, and sulphuric acid (H_2_SO_4_) 1 N were purchased from Fisher Chemical™ part of Thermo Fisher Scientific (Waltham, MA, USA).

### 3.2. Raw Materials

The production of the saponified substance involved the incorporation of multiple raw materials, which are listed below:

- Buffalo and cow milk were collected from “La Bufalara”, a company located in Lesina (Foggia, Italy). Both buffalo and cows were farmed through extensive grazing, which means that all animals were free to pasture and had free access to fresh grass and water;
- Extra virgin olive oil obtained from *Olea europaea* L. was purchased from a local company named “Antonio Nicola Vocino”, located at San Paolo di Civitate (Foggia, Italy), and it was produced in October 2023;
- Coconut oil was an organic virgin coconut oil, raw and cold-pressed, 100% organic (NaturaleBio, Rome, Italy), and was purchased at a local market;
- Lavender flowers (*Lavandula angustifolia*) and Thyme plants (*Thymus citrodorius*) were purchased from “Società M2 Energia”, a company located in San Severo (Foggia, Italy). The Lavender flowers were collected when they were fully bloomed but not completely mature, and then only the best flowers were selected. Leaves from Thyme plants were green and mature, and the flowers had not bloomed. Thyme and Lavender were grown under renewable energy systems, in particular photovoltaic panels;
- Grape pomace (var. Montepulciano) was obtained by a local winery (Antica Cantina, San Severo, Foggia, Puglia) in the production year 2022, and it was stored at −20 °C to avoid the enzymatic degradation of the polyphenols until use.

All vegetable samples were used in three different ways for the production of soaps:

1. Fresh, without any treatment (F);
2. Dried, at 30 °C until constant weight (for about 30 h) for Thyme and Lavender flowers, and for grape pomace, at 70 °C for 18 h (D);
3. Freeze-dried, after the application of a patented, non-invasive, physical technology (Patent n. 0001426984) (P) using a freeze-drier LIOSMART 8/5P, supplied by 5Pascal (Trezzano Sul Naviglio—Milano, Italy).

### 3.3. Raw Materials Analysis

The quality of buffalo and cow milk and extra virgin olive oil was defined through specific characterization analyses. For extra virgin olive oil peroxide content, acidity and spectrophotometric analyses (λ 232, 270, 276, and ∆K are specific ultraviolet spectrophotometric absorption parameters used to determine the quality, freshness, and purity of olive oil) were performed by following the European Regulations (GU CEE L. 248 5 September 1991 All. III and GU CEE L22 30 January 1993). Cow and buffalo milk samples were analyzed with the MilkoScan Ft 6000 (Foss Electric) to assess fat, protein, lactose, and casein contents [57].

The chemical characterization of the vegetable materials was conducted following the administration of the three distinct pre-treatments in order to discriminate between the three formulations: fresh, dried, and freeze-dried. The analyses conducted on vegetable materials were pH, moisture content, total phenolic content (TPC), DPPH radical scavenging activity, and polyphenol determination by high-performance liquid chromatography. To ensure the accurate and total execution of the analyses of TPC, DPPH, and polyphenol determination, all samples were subjected to methanolic extraction. The methanolic extraction of the phenolic compounds from plants and vegetables was performed according to [58] with slight modifications. Samples (0.5 g) were weighed and extracted with 20 mL of methanol (80%) acidified with 1% of hydrochloric acid (*v*/*v*), vortexed, sonicated in a cold sonicator bath for 30 min, and left at room temperature on a shaker for 1 h. After that, samples were left at room temperature for about 15 min and then centrifuged at 7500× *g* for 10 min. The above-mentioned method was carried out in triplicate, and all the supernatants were collected in a 20 mL flask. All the samples were stored at −20 °C until the analysis. All the analyses were performed in triplicate, and the results were expressed as mean (*n* = 3) ± standard deviation.

#### 3.3.1. pH Determination

The pH was assessed by following the procedures described in [59] with slight modifications. Briefly, samples (1 g) (Lavender, Thyme, and Grape pomace) were mixed into 50 mL of water and left to soak for 24 h before the pH determination. A pH meter (GLP 22-Crison Instruments, Barcelona (Spain)) calibrated between 4.00 and 9.21 was used for the pH determination.

#### 3.3.2. Moisture Content

The moisture content was assessed using the oven-dry method (80 °C), according to [60]. More specifically, 5 g of each sample of soap was dried in the oven until reaching a constant weight. The moisture content was then determined using the following equation:

Moisture content (%) = [(W1 − W2)/(W1)] × 100(1)

where W1 = Soap sample and W2 = Dried soap sample.

An ISCO 900—RS232 oven was used to dry the vegetables and carry out the moisture analysis.

#### 3.3.3. Total Phenolic Content (TPC)

The total phenolic content was measured through the Folin–Ciocalteu method, as described by Barberis et al. [61], with slight modifications. The methanolic extracts (0.02 mL) were mixed with 0.1 mL of the Folin–Ciocalteu reagent diluted in a ratio of 1:10 with distilled water (*v*/*v*), and after 5 min, 0.08 mL of a sodium carbonate (Na_2_CO_3_) solution was added. Samples were stored at room temperature in the dark for 2 h; then, the absorbance was read at 750 nm using a spectrophotometer (Power Wave XS2, Biotek, Milan, Italy). Gallic acid was used to determine the standard curve (5–500 mg/L) with a R^2^ of 0.9986 and a regression equation of y = 0.005x + 0.259. The results were expressed as mg of gallic acid equivalents (GAE) per 100 g of samples. An UV–VIS spectrophotometer with a microplate with clear flat-bottom 96-well, 400 ul, was used for the determination of total phenolic content.

#### 3.3.4. DPPH Radical Scavenging Activity

The DPPH radical scavenging activity was analyzed by using the 2,2-diphenyl-1-picryl-hydrazyl (DPPH) method, according to the procedure described by Fan et al. [62] and la Gatta et al. [63]. Trolox was used to determine the standard curve (5–500 mg/L) with a R^2^ of 0.9967 and a regression equation of y = 0.158x + 3.642. The results were expressed as μmol of TEAC (Trolox Equivalent Antioxidant Capacity) per 100 g of samples. An UV–VIS spectrophotometer (Power Wave XS2, Biotek, Milan, Italy) with a microplate with clear flat-bottom 96-well, 400 ul, was used for the determination of radical scavenging activity.

#### 3.3.5. Polyphenol Determination by High-Performance Liquid Chromatography (HPLC)

A liquid chromatograph Agilent 1200 Series system (Santa Clara, CA, USA) equipped with a Multospher 100 RP 18 column (5 μm, 125 × 4.6 mm, Chromatographie-Service GmbH, Langerwehe, Germania) set at 20 °C was used to separate and quantify the polyphenols. The polyphenol determination was performed as described by Ahmad-Qasem et al. [64] with some modifications. Separations were carried out by using a flow rate of 0.5 mL min^−1^, and the detection was set at 280 nm. The gradient separation was achieved by using the following mobile phases: (A) acetonitrile and (B) ultra-pure water containing 25 mL/L of glacial acetic acid; the gradient started from 80% of phase B and ended at 45% of phase B in 40 min, and the column was conditionate for 10 min between each run, so the chromatographic run lasted 50 min. Before the analysis, 1.5 mL of methanolic extract of the samples was dried and resuspended in 1 mL of mobile phase B, and an aliquot (100 μL) was injected into the column. Identification and quantitative analysis were performed according to the external standard method on the basis of a calibration curve obtained by the injection of the following standards at different concentrations: procyanidin B1, gallic acid, procyanidin B2, catechin, epicatechin, 4-hydroxybenzoic acid, syringic acid, p-coumaric acid, and quercetin. The calibration curves used were constructed by analyzing progressive dilutions of the standards (0–500 mg/L), whose parameters are shown in Table S1 (Supplementary Materials).

### 3.4. Soap Formulation

The soaps were made by following the procedures described in [13], with slight modifications. The formulations of soaps and milk-based soaps with the addition of the officinal plants (Lavender flowers and Thyme plant) and vegetables (Grape pomace) are summarized in Table 5.

**Figure 6:**
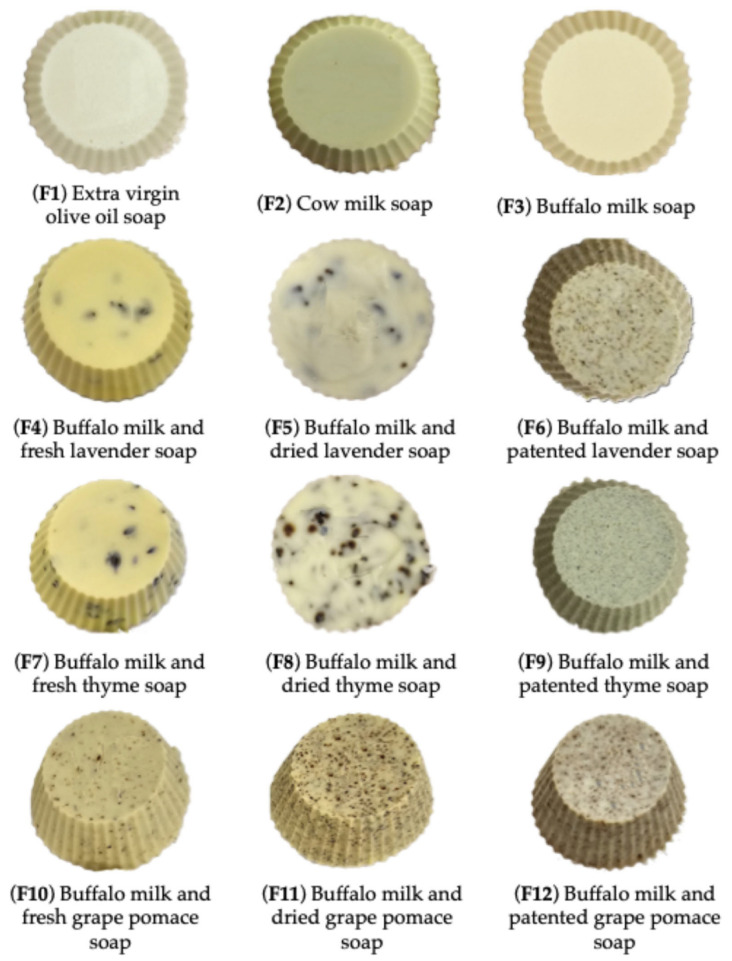
Artisanal soaps. (**F1**): soap made with extra virgin olive oil, coconut oil, and only water without replacing a part of it with milk; (**F2**): soap made with extra virgin olive oil, coconut oil, water, and cow milk; (**F3**): soap made with extra virgin olive oil, coconut oil, water, and buffalo milk; (**F4**): soap made with extra virgin olive oil, coconut oil, water, buffalo milk, and 3 g of fresh Lavender; (**F5**): soap made with extra virgin olive oil, coconut oil, water, buffalo milk, and 3 g of dried Lavender; (**F6**): soap made with extra virgin olive oil, coconut oil, water, buffalo milk, and 3 g of patented Lavender; (**F7**): soap made with extra virgin olive oil, coconut oil, water, buffalo milk, and 3 g of fresh Thyme; (**F8**): soap made with extra virgin olive oil, coconut oil, water, buffalo milk, and 3 g of dried Thyme; (**F9**): soap made with extra virgin olive oil, coconut oil, water, buffalo milk, and 3 g of patented Thyme; (**F10**): soap made with extra virgin olive oil, coconut oil, water, buffalo milk, and 3 g of fresh Grape pomace; (**F11**): soap made with extra virgin olive oil, coconut oil, water, buffalo milk, and 3 g of dried Grape pomace; and (**F12**): soap made with extra virgin olive oil, coconut oil, water, buffalo milk, and 3 g of patented Grape pomace.

All soaps were produced with natural ingredients, chemically characterized and produced according to the following procedure: 225 g of extra virgin olive oil and 12.5 g of coconut oil were slowly heated up to 37 °C; 30.25 g of NaOH was dissolved in 17.5 g of water (25%) and 50 g of milk (75%) and left to wait for the temperature to reach 37 °C (milk was replaced with water for the olive oil soap). The NaOH solution was added to the oils’ solution by stirring constantly until the mixture was visibly thickening. The soap was then placed into single portion molds (50 g) and left fallow for 1 day before unmolding (Figure 6). The maturation process of the soap took around six weeks.

The soaps produced are listed and reported in Table 5. Briefly, soap made with extra virgin olive oil, coconut oil, and only water without replacing a part of it with milk represents (**F1**); soap made with extra virgin olive oil, coconut oil, water, and cow milk represents (**F2**); soap made with extra virgin olive oil, coconut oil, water, and buffalo milk represents (**F3**); soap made with extra virgin olive oil, coconut oil, water, buffalo milk, and 3 g of fresh/dried or patented Lavender represents (**F4**, **F5**, and **F6**); soap made with extra virgin olive oil, coconut oil, water, buffalo milk, and 3 g of fresh/dried or patented Thyme represents (**F7**, **F8**, and **F9**); and soap made with extra virgin olive oil, coconut oil, water, buffalo milk, and 3 g of fresh/dried or patented Grape pomace represents (**F10**, **11**, and **12**).

### 3.5. Soap Analysis

To obtain a characterization of the experimental artisanal soaps, a series of chemical analyses was conducted. For the analysis of bio compounds (TPC and DPPH analyses), beyond the methanolic extraction, water extraction was conducted to simulate the real passage of active compounds during use. The method used was the same procedure applied in the methanolic extraction as described in the previous Section 3.3.

All the analyses were performed in triplicate, and the results were expressed as mean (*n* = 3) ± standard deviation.

#### 3.5.1. pH Determination

The pH determination was assessed by following the procedures described in [65] with slight modifications. In detail, 150 mg of soap samples were homogenized with 15 mL of deionized water without producing much lather, and then, the samples were left for 24 h before the pH determination.

#### 3.5.2. Moisture Content

The moisture content was determined as described in the previous Section 3.3.2.

#### 3.5.3. Total Phenolic Content (TPC)

The total phenolic content (TPC) was determined as described in the previous Section 3.3.3. on the methanolic and water extracts.

#### 3.5.4. DPPH Radical Scavenging Activity

The DPPH radical scavenging activity was determined as described in the previous Section 3.3.4. on the methanolic and water extracts.

#### 3.5.5. Polyphenol Determination by High-Performance Liquid Chromatography (HPLC)

The polyphenol determination by high-performance liquid chromatography (HPLC) was performed as described in the previous Section 3.3.5.

#### 3.5.6. Soaps’ Insoluble Matter

The soap’s insoluble matter was determined following the method described by Idoko et al. [39] with slight modifications. In particular, 5 g of soap samples were mixed with 100 mL of hot ethanol and transferred into a pre-weighed filter paper. The residue was dried at 105 °C for 30 min, cooled, and then weighed. The insoluble matter in alcohol was then determined by using the following equation:

MIA (%) = [(Ws − WP)/(ms)] × 100(2)

where Ws = weight of the filter paper + soap sample, WP = filter paper weight, and ms = mass of soap sample.

#### 3.5.7. Soaps’ Total Free Fatty Matter

Total free fatty matter was obtained through the application of the following equation:

TFFM = [100 − (MC − MIA)]/1.085(3)

where MC = Moisture Content, and MIA = Matter insoluble in alcohol, in agreement with Idoko et al. [39].

#### 3.5.8. Total Alkali Content

The total alkali content was determined through an acid–base titration method, as described by Zayed et al., 2024 [44]. In detail, 10 g of soap was dissolved in ethanol and then mixed with 5 mL H_2_SO_4_ 1 N, achieving a pH value of about 1.10. The excess acid was titrated with a solution of NaOH 1 N with phenolphthalein as an indicator. The total alkali content was calculated using the following equation:

Total Alkali content (%) = (Vacid − Vbase)/(msample) × 3.1(4)

where Vacid = volume of H_2_SO_4_, Vbase = volume of NaOH, and msample = mass in gram of sample.

### 3.6. Sensory Soaps Evaluation

Sensory analysis was carried out in March 2024 at the University of Foggia, Department of Agricultural Sciences, Food, Natural Resources and Engineering (DAFNE) by 50 judges (men and women, 20–55 years old). Soap samples were evaluated during a single session for a group of five panelists (in total, 10 sessions) that took place at the Sensory Laboratory of the University of Foggia.

The participants were briefed about the test’s procedure. In the first phase, participants evaluated the samples based on the general aspect and smell. Afterwards, every panelist washed their hands with soap and maintained contact with their hands for 2 min and then washed with water for 2 min. In order to avoid sensibilization, 5 min breaks were considered between every soap evaluation. Each sample was assigned a random number code and presented to the participants in an identical container and portioned equally in weight and shape.

For each soap sample, three parameters were evaluated: general aspect, smell of the hands after use, and softness of the skin after use. Sensory attributes were evaluated using a five-point (1–5) hedonic scale ranging from extremely unpleasant (1) to extremely pleasant (5). Verbal informed consent was obtained from all participants for their participation in the sensorial analysis and the publication of this study, which was a pre-commercial and non-therapeutic test. Written consent was not required according to “REGOLAMENTO (UE) 2016/679 DEL PARLAMENTO EUROPEO E DEL CONSIGLIO del 27 April 2016, Article 4, comma 11”, which exempts this type of study from ethical approval [66].

### 3.7. Statistical Analysis

One-way ANOVA test was used to evaluate the effects of the addition of plants/vegetables on the evaluated features of the samples, and their differences were evaluated by Tukey’s test using XLSTAT 2024.1.0 software (Addinsoft, New York, NY, USA).

## 4. Conclusions

Generally, consumers are persuaded by the use of natural raw materials, considering their higher quality. The different artisanal solid soap formulations studied in this experimentation were assessed by using natural ingredients, including odorants, to generate greater consumer interest in the product. The soap bars were artisanal, and they were tested in order to obtain an efficient and pleasant soap for daily use. The performed analyses led us to define the composition of the raw materials (cow and buffalo milk), the chemical parameters of fresh, dried, and patented plants/vegetables, and the chemical parameters of the resulting soap bars. The obtained results revealed that the total polyphenolic content was not directly related to the antioxidant activity, but we implied that the best antioxidant activity values were due to the content of flavonoids, as demonstrated by the methanolic extracts, because of their greater solubility in this solvent. All the soap formulations showed low moisture percentage and totally fatty matter values, high matter insoluble in alcohol, an alkaline pH, and a lower value of total alkali. According to the literature, a low moisture percentage prolongs shelf-life, preventing bacterial growth, and an alkaline pH value (about 10) permits the use on skin, indicating a high cleansing power and lathering, but it could be strong for dry skin; therefore, the tested formulations need to be improved. Totally fatty matter and matter insoluble in alcohol showed acceptable values for daily hygiene. Therefore, the data obtained following the quantification of insoluble fatty matter and totally fatty matter reflect the possibility of improving the saponification process, so as to limit any portions of non-fat solid substances present in the milk, considering the purpose of the final solid soap too. Mostly, the tested twelve soap formulations showed an acceptable quality for safe use. Moreover, it must be considered that the amphipathic properties of milk protein and fatty acids with short chains affect skin softness, increasing the milk soap quality. The addition of the plants/vegetables changed the chemical properties of the soaps, and, considering also the sensory quality, the best formulations were those related to the use of, firstly, dried and patented Lavender, followed by Grape pomace soaps. In conclusion, the data obtained have demonstrated that the utilization of buffalo milk in combination with vegetables represents a valid alternative to the use of surplus milk.

## 5. Patents

This manuscript reports the use of the patent n. 0001426984.

## Figures and Tables

**Table 1:** Olive oil qualitative analysis.

	Method	Results	Legal Limits Reg. (UE) 1830/15
Acidity (%)	GU CEE L248 5 September 1991 All III	0.197 ± 0.003	<0.8
Peroxides (meq/kg)	GU CEE L248 5 September 1991 All III	9.160 ± 0.052	<20
λ 232	GU CEE L22 30 January 1993	1.763 ± 0.091	<2.50
λ 270	GU CEE L22 30 January 1993	0.102 ± 0.005	<0.220
λ 276	GU CEE L22 30 January 1993	0.102 ± 0.005	-
ΔK	GU CEE L22 30 January 1993	−0.004 ± 0.001	<0.01

**Table 2:** Buffalo and cow milk compositions determined by Milkoscan. Results were expressed as mean ± SD of three repetitions.

	Fat (%)	Protein (%)	Lactose (%)	Casein (%)
Buffalo milk	7.96 ± 0.31 ^a^	4.62 ± 0.02 ^a^	4.78 ± 0.01 ^a^	3.57 ± 0.04 ^a^
Cow milk	4.88 ± 0.80 ^b^	3.77 ± 0.02 ^b^	4.65 ± 0.03 ^b^	2.91 ± 0.03 ^b^

Significant differences (*p* < 0.05) are expressed with different superscript letters within a column for each parameter.

**Table 3:** Chemical characterization of the raw materials used for the preparation of solid soaps. Results were expressed as mean (*n* = 3) ± SD.

Samples	pH (UpH)	Moisture (%)	Total Polyphenol Content (mg of Gallic Acid/100 g)	Antioxidant Activity (µmol TEAC/100 g)
Fresh Lavender	5.47 ± 0.09 ^e^	63.5 ± 0.7 ^b^	394 ± 2 ^i^	1265 ± 3 ^d^
Dried Lavender	5.78 ± 0.15 ^c,d^	5.16 ± 0.5 ^d^	1572 ± 2 ^f^	1366 ± 9 ^b^
Patented Lavender	5.62 ± 0.04 ^d,e^	3.05 ± 0.3 ^f^	9851 ± 1 ^c^	1282 ± 6 ^d^
Fresh Thyme	5.91 ± 0.01 ^b,c^	51.7 ± 2.5 ^c^	546 ± 2 ^h^	1063 ± 10 ^g^
Dried Thyme	6.01 ± 0.01 ^b^	5.06 ± 0.6 ^d^	1032 ± 1 ^g^	1285 ± 7 ^d^
Patented Thyme	6.41 ± 0.01 ^a^	2.11 ± 0.5 ^g^	11,967 ± 2 ^b^	1193 ± 5 ^f^
Fresh Grape Pomace	3.40 ± 0.05 ^f^	82.5 ± 2.3 ^a^	3032 ± 2 ^e^	1235 ± 10 ^e^
Dried Grape Pomace	3.54 ± 0.06 ^f^	6.12 ± 1.1 ^d^	7745 ± 1 ^d^	1312 ± 8 ^c^
Patented Grape Pomace	3.53 ± 0.03 ^f^	4.01 ± 0.4 ^e^	18,316 ± 1 ^a^	1524 ± 4 ^a^

^a, b, c,…….i^ (*p* < 0.05) significant differences are expressed with different superscript letters within a column for each parameter.

**Table 4:** Chemical characterization of solid soaps. Results were expressed as mean (*n* = 3) ± SD.

Samples	pH (UpH)	Moisture (%)	Insoluble Fatty Matter (%)	Totally Fatty Matter (%)	Total Alkali (%)	TPC in MeOH (mg Gallic Acid /100 g)	TPC in Water (mg Gallic Acid /100 g)	Antioxidant Activity in MeOH (µmol Teac/100 g)	Antioxidant Activity in Water (µmol Teac/100 g)
**F1**	10.12 ± 0.02 ^f^	4.20 ± 0.4 ^h,i^	55.17 ± 0.25 ^a^	45.19 ± 0.63 ^a,b^	0.159 ± 0.26 ^c^	187 ± 1 ^k^	537 ± 0.4 ^i^	1318 ± 7 ^d^	715 ± 5 ^g^
**F2**	10.84 ± 0.04 ^a^	6.43 ± 0.2 ^b,c,d^	61.15 ± 4.3 ^a^	41.73 ± 4.11 ^a,b^	0. 348 ± 0.33 ^b^	243 ± 1 ^i^	558 ± 0.3 ^h^	1114 ± 3 ^i^	482 ± 5 ^h^
**F3**	10.44 ± 0.01 ^b,c,d,e^	5.18 ± 0.06 ^f,g^	56.97 ± 2.6 ^a^	44.43 ± 2.4 ^a,b^	0.418 ± 0.25 ^a^	252 ± 1 ^h^	639 ± 1 ^f^	1511 ± 2 ^b^	883 ± 5 ^b^
**F4**	10.49 ± 0.07 ^c,d,e^	5.47 ± 0.3 ^e,f,g^	56.91 ± 3.2 ^a^	44.75 ± 3.3 ^a,b^	0.355 ± 0.41 ^b^	255 ± 1 ^g^	457 ± 1 ^j^	1412 ± 2 ^c^	818 ± 5 ^d^
**F5**	10.72 ± 0.11 ^a,b,c^	6.21 ± 0.08 ^c,d,e^	56.13 ± 1.6 ^a^	46.16 ± 1.6 ^a,b^	0.342 ± 0.39 ^b^	307 ± 1 ^b^	739 ± 1 ^d^	1231 ± 10 ^f^	788 ± 1 ^e^
**F6**	10.46 ± 0.04 ^d,e^	7.05 ± 0.23 ^a,b^	53.20 ± 3.1 ^a^	49.63 ± 3.04 ^a,b^	0.331 ± 0.44 ^b^	298 ± 1 ^c^	666 ± 0.4 ^e^	1206 ± 3 ^g^	N.D.
**F7**	10.37 ± 0.07 ^e,f^	3.76 ± 0.1 ^i^	56.70 ± 2.02 ^a^	40.65 ± 1.8 ^b^	0.362 ± 0.35 ^a,b^	285 ± 1 ^e^	762 ± 0.5 ^b^	1573 ± 3 ^a^	729 ± 3 ^f^
**F8**	10.82 ± 0.07 ^a,b^	4.75 ± 0.2 ^g,h^	54.60 ± 1.45 ^a^	46.30 ± 1.5 ^a,b^	0.360 ± 0.39 ^a,b^	285 ± 1 ^e^	765 ± 0.2 ^a^	1149 ± 9 ^h^	842 ± 3 ^c^
**F9**	10.52 ± 0.08 ^c,d,e^	6.12 ± 0.06 ^c,d,e^	51.39 ± 0.6 ^a^	50.50 ± 0.6 ^a^	0.356 ± 0.41 ^b^	275 ± 1 ^f^	566 ± 0.1 ^g^	1135 ± 2 ^h^	N.D.
**F10**	10.12 ± 0.07 ^f^	7.28 ± 0.06 ^a^	52.2 ± 3.8 ^a^	50.80 ± 3.4 ^a^	0.329 ± 0.33 ^b^	227 ± 0.2 ^j^	745 ± 0.2 ^c^	1038 ± 7 ^j^	842 ± 2 ^c^
**F11**	10.34 ± 0.1 ^e,f^	6.93 ± 0.26 ^a,b,c^	55.8 ± 1.8 ^a^	47.12 ± 1.44 ^a,b^	0.321 ± 0.29 ^b^	289 ± 1 ^d^	351 ± 1 ^l^	1136 ± 5 ^h^	958 ± 3 ^a^
**F12**	10.66 ± 0.01 ^a,b,c^	5.69 ± 0.08 ^d,e,f^	53.06 ± 2.1 ^a^	48.50 ± 1.8 ^a,b^	0.311 ± 0.48 ^b^	383 ± 1 ^a^	383 ± 1 ^k^	1266 ± 8 ^e^	214 ± 2 ^i^

Significant differences (*p* < 0.05) are expressed with different superscript letters within a column for each parameter. N.D.: Not Detected.

**Table 5:** Ingredients and their amounts (g) used in the soap’s formulations. EVO = Extra Virgin Olive oil; F = Fresh; HD = Heat Dried; P = Patent.

Soap Formulation	EVO	Coconut Oil	Cow Milk	Buffalo Milk	F	HD	P	F	HD	P	F	HD	P	Water	NaOH
					Lavender			Thyme			Grape pomace				
**F1**	225	12.5	-	-	-	-	-	-	-	-	-	-	-	67.5	30.25
**F2**	225	12.5	50		-	-	-	-	-	-	-	-	-	17.5	30.25
**F3**	225	12.5	-	50										17.5	30.25
**F4**	225	12.5	-	50	3	-	-	-	-	-	-	-	-	17.5	30.25
**F5**	225	12.5	-	50	-	3	-	-	-	-	-	-	-	17.5	30.25
**F6**	225	12.5	-	50	-	-	3	-	-	-	-	-	-	17.5	30.25
**F7**	225	12.5	-	50	-	-	-	3	-	-	-	-	-	17.5	30.25
**F8**	225	12.5	-	50	-	-	-	-	3	-	-	-	-	17.5	30.25
**F9**	225	12.5	-	50	-	-	-	-	-	3	-	-	-	17.5	30.25
**F10**	225	12.5	-	50	-	-	-	-	-	-	3	-	-	17.5	30.25
**F11**	225	12.5	-	50	-	-	-	-	-	-	-	3	-	17.5	30.25
**F12**	225	12.5	-	50	-	-	-	-	-	-	-	-	3	17.5	30.25

## Data Availability

The raw data supporting the conclusions of this article will be made available by the authors on request.

## Supplementary Materials

The following supporting information can be downloaded at: https://www.mdpi.com/article/10.3390/molecules31040734/s1, Table S1: Linearity, LOD, and LOQ of the chromatographic method for the determination of Standards.

## References

[1] Zicarelli L., Presicce G.A. (2017). Influence of seasonality on buffalo production. The Buffalo (Bubalus bubalis) Production and Research.

[2] van Dijk K., van Dijk K., Taylor J.G. (2011). Soap is the onset of civilization. Cleanliness and Culture, Indonesian Histories.

[3] Kailasapathy K. (2015). Chemical Composition, Physical, and Functional Properties of Milk and Milk Ingredients. Dairy Processing and Quality Assurance.

[4] Faria-Silva C., Ascenso A., Costa A.M., Marto J., Carvalheiro M., Ribeiro H.M., Simões S. (2020). Feeding the skin: A new trend in food and cosmetics convergence. Trends Food Sci. Technol..

[5] Wu X., Shen J., Zhong Y., Zhao X., Zhou W., Gao P., Wang X., An W. (2024). Large-Scale Isolation of Milk Exosomes for Skincare. Pharmaceutics.

[6] Susanti A.D., Saputro S., Wibowo W.A. (2018). Optimization of Cow’s Milk Processing into Milk Soap Bar on Small-Medium-Micro Enterprises (UMKM). Equilib. J. Chem. Eng..

[7] Murti T.W., Walida I., Rayi A., Pradana M.W.E., Wisudanta G.M. (2024). Physico-chemical charateristics of goat’s milk soap. 10th International Seminar on Tropical Animal Production, IOP Conference Series: Earth and Environmental Science.

[8] Abd El-Salam M.H., El-Shibiny S. (2011). A comprehensive review on the composition and properties of buffalo milk. Dairy Sci. Technol..

[9] Jones G., Jones G. (2010). Scent and Paris. Beauty Imagination. A History of the Global Beauty Industry.

[10] Ossai E.K. (2014). Preparation and Characterization of Metal Soaps of Cocos Nucifera Seed Oil. J. Appl. Sci. Environ. Manag..

[11] Antignac E., Nohynek G.J., Re T., Clouzeau J., Toutain H. (2011). Safety of botanical ingredients in personal care products/cosmetics. Food Chem. Toxicol..

[12] Hayati S.N., Rosyida V.T., Darsih C., Nisa K., Indrianingsih A.W., Apriyana W., Ratih D. (2020). Physicochemical properties, antimicrobial and antioxidant activity of ganoderma transparent soap. IOP Conference Series: Earth and Environmental Science.

[13] Félix S., Araújo J., Pires A.M., Sousa A.C. (2017). Soap production: A green prospective. Waste Manag..

[14] Aremu M.O., Ibrahim H., Bamidele T.O. (2015). Physicochemical characteristics of the oils extracted from some Nigerian plant foods—A review. Chem. Process Eng. Res..

[15] Kapadiya D.B., Prajapati D.B., Jain A.K., Mehta B.M., Darji V.B., Aparnathi K.D. (2016). Comparison of Surti goat milk with cow and buffalo milk for gross composition, nitrogen distribution, and selected minerals content. Vet. World.

[16] Cotte J. (1991). Le lait, une matière d’avenir pour la cosmétique. Le Lait.

[17] Ribeiro A.C., Ribeiro S.D.A. (2010). Specialty products made from goat milk. Small Rumin. Res..

[18] Samman S., Chow J.W.Y., Foster M.J., Ahmad Z.I., Phuyal J.L., Petocz P. (2008). Fatty acid composition of edible oils derived from certified organic and conventional agricultural methods. Food Chem..

[19] Kähkönen M.P., Hopia A.I., Vuorela H.J., Rauha J.P., Pihlaja K., Kujala T.S., Heinonen M. (1999). Antioxidant activity of plant extracts containing phenolic compounds. J. Agric. Food Chem..

[20] la Gatta B., Liberatore M.T., Dilucia F., Rutigliano M., Baiano A., Di Luccia A., Turco F., Turco D. (2024). Study of Ready-to-eat Omelette enriched with dried and freeze-dried vegetables. Food Biosci..

[21] Nicoli M.C., Anese M., Parpinel M. (1999). Influence of processing on the antioxidant properties of fruit and vegetables. Trends Food Sci. Technol..

[22] Fernández-Pachón M.S., Villaño D., García-Parrilla M.C., Troncoso A.M. (2004). Antioxidant activity of wines and relation with their polyphenolic composition. Anal. Chim. Acta.

[23] Gregoris E., Stevanato R. (2010). Correlations between polyphenolic composition and antioxidant activity of Venetian propolis. Food Chem. Toxicol..

[24] Liberatore M.T., Dilucia F., Rutigliano M., Viscecchia R., Spano G., Capozzi V., Bimbo F., Di Luccia A., la Gatta B. (2025). Polyphenolic characterization, nutritional and microbiological assessment of newly formulated semolina fresh pasta fortified with grape pomace. Food Chem..

[25] Lambers H., Piessens S., Bloem A., Pronk H., Finkel P. (2006). Natural skin surface pH is on average below 5, which is beneficial for its resident flora. Int. J. Cosmet. Sci..

[26] Segger D., Aßmus U., Brock M., Erasmy J., Finkel P., Fitzner A., Heuss H., Kortemeier S., Munke S., Rheinländer T. (2008). Multicenter Study on Measurement of the Natural pH of the Skin Surface. Int. J. Cosmet. Sci..

[27] Öhman H., Vahlquist A. (1994). In vivo studies concerning a pH gradient in human stratum corneum and upper epidermis. Acta Derm. Venereol..

[28] Fukuda K., Ito Y., Furuichi Y., Matsui T., Horikawa H., Miyano T., Okada T., van Logtestijn M., Tanaka R.J., Miyawaki A. (2024). Three stepwise pH progressions in stratum corneum for homeostatic maintenance of the skin. Nat. Commun..

[29] Widyasanti A., Ayuningtyas B., Rosalinda S. (2020). Characterization of liquid soap from castor oil (*Ricinus communis*) with the addition of white tea extracts. IOP Conference Series: Earth and Environmental Science.

[30] Ali S.M., Yosipovitch G. (2013). Skin pH: From Basic Science to Basic Skin Care. Acta Derm. Venereol..

[31] Al-Hiyaly S.A.K., Al-Tamimi J.A., Al-Tai S.R.K. (2014). Assessment of lead contamination in Aleppo soaps available in Iraqi markets. J. Genet. Environ. Resour. Conserv..

[32] Fluhr J.W., Kao J., Ahn S.K., Feingold K.R., Elias P.M., Jain M. (2001). Generation of free fatty acids from phospholipids regulates stratum corneum acidification and integrity. J. Investig. Dermatol..

[33] Wickett R.R., Trobaugh C.M., Wax S.J. (1990). Personal care products: Effects on skin surface, pH. Cosmet. Toilet..

[34] Lukić M., Pantelić I., Savić S.D. (2021). Towards optimal pH of the skin and topical formulations: From the current state of the art to tailored products. Cosmetics.

[35] (2007). Encyclopedia of Industrial Chemical Analysis.

[36] Tewari K.S. (2004). A Textbook of Chemistry.

[37] Mahesar S.A., Chohan R., Sherazi S.T.H. (2019). Evaluation of physico-chemical properties in Selected Branded soaps. Pak. J. Anal. Environ. Chem..

[38] Ogunsuyi H.O., Akinnawo C.A. (2012). Quality assessment of soaps produced from palm bunch ash-derived alkali and coconut oil. J. Appl. Sci. Environ. Manag..

[39] Idoko O., Emmanuel S.A., Salau A.A., Obigwa P.A. (2018). Quality assessment on some soaps sold in Nigeria. Niger. J. Technol..

[40] (1975). The International Standard Specification for Soaps.

[41] Betsy K.J., Jilu M., Fathima R., Varkey J.T. (2013). Determination of Alkali Content & Total Fatty Matter in Cleansing Agents. Asian J. Sci. Appl. Technol..

[42] Vivian O.P., Nathan O., Osano A., Mesopirr L., Omwoyo W.N. (2014). Assessment of the Physicochemical Properties of Selected Commercial Soaps Manufactured and Sold in Kenya. Open J. Appl. Sci..

[43] Roila A., Salmiah A., Ghazali R. (2001). Properties of Sodium Soap Derived from Palm-Based Dihydroxystearic Acid. J. Oil Palm Res..

[44] Zayed L., Gablo N., Kalcakova L., Dordevic S., Kushkevych I., Dordevic D., Tremlova B. (2024). Utilizing used cooking oil and organic waste: A sustainable approach to soap production. Processes.

[45] Sik B., Buzás H., Kapcsándi V., Lakatos E., Daróczi F., Székelyhidi R. (2023). Antioxidant and polyphenol content of different milk and dairy products. J. King Saud Univ.-Sci..

[46] Marina A.M., Che Man Y.B., Nazimah S.A.H., Amin I. (2009). Antioxidant capacity and phenolic acids of virgin coconut oil. Int. J. Food Sci. Nutr..

[47] Servili M., Sordini B., Esposto S., Urbani S., Veneziani G., Di Maio I., Selvaggini R., Taticchi A. (2014). Biological Activities of Phenolic Compounds of Extra Virgin Olive Oil. Antioxidants.

[48] Zain S.N.D.M., Omar W.A.W. (2018). Antioxidant Activity, Total Phenolic Content and Total Flavonoid Content of Water and Methanol Extracts of Phyllanthus species from Malaysia. Pharmacogn. J..

[49] Lim Y.Y., Murtijaya J. (2007). Antioxidant properties of Phyllanthus amarus extracts as affected by different drying methods. LWT-Food Sci. Technol..

[50] Markom M., Hasan M., Daud W.R.W., Singh H., Jahim J.M. (2007). Extraction of hydrolysable tannins from *Phyllanthus niruri* Linn.: Effects of solvents and extraction methods. Sep. Purif. Technol..

[51] Poh-Hwa T., Yoke-Kqueen C., Indu Bala J., Son R. (2011). Bioprotective properties of three Malaysia Phyllanthus species: An investigation of the antioxidant and antimicrobial activities. Int. Food Res. J..

[52] Wong B.Y., Tan C.P., Ho C.W. (2013). Effect of solid-to-solvent ratio on phenolic content and antioxidant capacities of “Dukung Anak” (*Phyllanthus niruri*). Int. Food Res. J..

[53] Tiwari U., Cummins E. (2013). Factors influencing levels of phytochemicals in selected fruit and vegetables during pre-and post-harvest food processing operations. Food Res. Int..

[54] Phan M.A.T., Paterson J., Bucknall M., Arcot J. (2018). Interactions between phytochemicals from fruits and vegetables: Effects on bioactivities and bioavailability. Crit. Rev. Food Sci. Nutr..

[55] Gbylik-Sikorska M., Gajda A., Burmańczuk A., Grabowski T., Posyniak A. (2019). Development of a UHPLC-MS/MS method for the determination of quercetin in milk and its application to a pharmacokinetic study. J. Vet. Res..

[56] Cairone F., Petralito S., Scipione L., Cesa S. (2021). Study on Extra Virgin Olive Oil:Quality Evaluation by Anti-Radical Activity, Color Analysis, and Polyphenolic HPLC-DAD Analysis. Foods.

[57] Sánchez A., Sierra D., Luengo C., Corrales J.C., De La Fe C., Morales C.T., Gonzalo C. (2007). Evaluation of the MilkoScan FT 6000 milk analyzer for determining the freezing point of goat’s milk under different analytical conditions. J. Dairy Sci..

[58] Turgay O., Esen Y. (2015). Antioxidant, total phenolic and antimicrobial characteristics of some species. Bulg. J. Agric. Sci..

[59] Karabagias I.K., Karabagias V.K., Riganakos K.A. (2019). Physico-chemical parameters, phenolic profile, in vitro antioxidant activity and volatile compounds of ladastacho (*Lavandula stoechas*) from the region of Saidona. Antioxidants.

[60] Oyekunle J.A., Ore O.T., Ogunjumelo O.H., Akanni M.S. (2021). Comparative chemical analysis of Indigenous Nigerian soaps with conventional ones. Heliyon.

[61] Barberis A., Cefola M., Pace B., Azara E., Spissu Y., Serra P.A., Logrieco A.F., D’hallewin G., Fadda A. (2019). Postharvest application of oxalic acid to preserve overall appearance and nutritional quality of fresh-cut green and purple asparagus during cold storage: A combined electrochemical and mass-spectrometry analysis approach. Postharvest Biol. Technol..

[62] Fan R., Yuan F., Wang N., Gao Y., Huang Y. (2015). Extraction and analysis of antioxidant compounds from the residues of *Asparagus officinalis* L. J. Food Sci. Technol..

[63] la Gatta B., Rutigliano M., Liberatore M.T., Dilucia F., Spadaccino G., Quinto M., Di Luccia A. (2023). Preservation of bioactive compounds occurring in fresh pasta fortified with artichoke bracts and tomato powders obtained with a novel pre-treatment. LWT.

[64] Ahmad-Qasem M.H., Barrajon-Catalan E., Micol V., Carcel J.A., Garcia-Perez J.V. (2013). Influence of air temperature on drying kinetics and antioxidant potential of olive pomace. J. Food Eng..

[65] Tarun J., Susan J., Suria J., Susan V.J., Criton S. (2014). Evaluation of pH of bathing soaps and shampoos for skin and hair care. Indian J. Dermatol..

[66] Europa (2016). Regolamento (UE) 2016/679 del Parlamento Europeo e del Consiglio del 27 Aprile 2016 Relativo Alla Protezione Delle Persone Fisiche con Riguardo al Trattamento dei Dati Personali, Nonché alla Libera Circolazione di Tali Dati e Che Abroga la Direttiva 95/46/CE (Regolamento Generale Sulla Protezione dei Dati).

