# Supplementary material for: Buffalo Milk: Alternative Use for Soap Preparation Enriched with Vegetables
Source: Molecules. 2026 Feb 20;31(4):734. doi: 10.3390/molecules31040734 (PMC12943392; doi:10.3390/molecules31040734)
Supplement: Supplementary file 1 [file molecules-31-00734-s001.zip › molecules-4141368-supplementary.pdf]

Table S1 - Linearity, LOD, and LOQ of the chromatographic method for the determination of Standards

Standards	Range, mg/L	R^2	LOD, mg/L	LOQ, mg/L
Procyanidin B1	0-500	0.9996	0.05	0.098
Gallic acid	0-500	0.9994	0.01	0.022
Procyanidin B2	0-500	0.9991	0.05	0.083
(+)-catechin	0-500	0.9993	0.05	0.186
(-)-epicatechin	0-500	0.9997	0.05	0.062
4-hydroxybenzoic acid	0-500	0.9996	0.02	0.054
Syringic acid	0-500	0.9997	0.01	0.027
p-coumaric acid	0-500	0.9995	0.01	0.025
Quercetin	0-500	0.9995	0.08	0.150
